# Quantitative insertion-site sequencing (QIseq) for high throughput phenotyping of transposon mutants

**DOI:** 10.1101/gr.200279.115

**Published:** 2016-07

**Authors:** Iraad F. Bronner, Thomas D. Otto, Min Zhang, Kenneth Udenze, Chengqi Wang, Michael A. Quail, Rays H.Y. Jiang, John H. Adams, Julian C. Rayner

**Affiliations:** 1 Malaria Programme, Wellcome Trust Sanger Institute, Genome Campus, Hinxton, Cambridgeshire CB10 1SA, United Kingdom;; 2 Center for Global Health and Infectious Diseases, Department of Global Health, University of South Florida, Tampa, Florida 33612, USA

## Abstract

Genetic screening using random transposon insertions has been a powerful tool for uncovering biology in prokaryotes, where whole-genome saturating screens have been performed in multiple organisms. In eukaryotes, such screens have proven more problematic, in part because of the lack of a sensitive and robust system for identifying transposon insertion sites. We here describe quantitative insertion-site sequencing, or QIseq, which uses custom library preparation and Illumina sequencing technology and is able to identify insertion sites from both the 5′ and 3′ ends of the transposon, providing an inbuilt level of validation. The approach was developed using *piggyBac* mutants in the human malaria parasite *Plasmodium falciparum* but should be applicable to many other eukaryotic genomes. QIseq proved accurate, confirming known sites in >100 mutants, and sensitive, identifying and monitoring sites over a >10,000-fold dynamic range of sequence counts. Applying QIseq to uncloned parasites shortly after transfections revealed multiple insertions in mixed populations and suggests that >4000 independent mutants could be generated from relatively modest scales of transfection, providing a clear pathway to genome-scale screens in *P. falciparum*. QIseq was also used to monitor the growth of pools of previously cloned mutants and reproducibly differentiated between deleterious and neutral mutations in competitive growth. Among the mutants with fitness defects was a mutant with a *piggyBac* insertion immediately upstream of the kelch protein K13 gene associated with artemisinin resistance, implying mutants in this gene may have competitive fitness costs. QIseq has the potential to enable the scale-up of *piggyBac-*mediated genetics across multiple eukaryotic systems.

Despite recent progress, malaria remains one of the most significant global public health burdens, with more than 300 million cases each year. The development and spread of resistance to current front line antimalarial drugs (Ashley et al. 2014) and the continued lack of an effective vaccine (malERA Consultative Group on Vaccines 2011) mean that systematic approaches to identify new intervention targets are needed now more than ever. Over the past 25 yr, experimental genetics has proven to be a powerful tool for understanding gene function and prioritizing targets in the major human malaria pathogen, *Plasmodium falciparum.* Genetic approaches have yielded significant breakthroughs in our understanding of parasite metabolism and pathogenesis (Ekland and Fidock 2007; Cowman et al. 2012), but almost all such studies have been carried out on a gene by gene reverse genetics basis and are necessarily limited in scale. The majority of the >5000 gene *P. falciparum* genome therefore remains unstudied using genetic tools.

New approaches are needed if large-scale functional genomics analysis is to become a reality. The development of new gene targeting approaches using site-specific cleavage to increase efficiency may be one such tool (Ghorbal et al. 2014; Wagner et al. 2014), but even here individual mutations are likely to be made one at a time. Transposon-mediated mutagenesis has the significant advantage of providing the opportunity to make large numbers of random mutations simultaneously in a pool of parasites and to phenotype them together, radically increasing the potential scale, and also allowing for unbiased genetic screens. In recent years, the *piggyBac* transposon has been used widely in multiple eukaryotic organisms to create gene disruptions, alter gene expression patterns, trap promoters, and generate gene fusions (Cary et al. 1989; Fraser et al. 1995; Gonzalez-Estevez et al. 2003; Thibault et al. 2004; Balu et al. 2005; Wu et al. 2006)*. piggyBac* inserts into TTAA target sequences, which are abundant in the AT-rich *P. falciparum* genome, and the *piggyBac* transposon has been used to generate more than 200 *P. falciparum* mutant lines, some of which have been followed up in detailed phenotyping studies (Balu et al. 2009, 2010; Fonager et al. 2011; Ikadai et al. 2013)*.* However, two critical obstacles have limited *piggyBac* mutagenesis approaches for *P. falciparum* genome-scale screening—the ability to identify transposon insertion sites at high efficiency and large scale, and a method to accurately phenotype large numbers of transposon mutants growing in parallel in a single pool.

We have applied Illumina-based next generation sequencing technologies to solve both of these problems. Several Illumina-based approaches for transposon insertion site identification have previously been developed for bacteria, including Tn-seq (van Opijnen et al. 2009), Tn-seq circle method (Gallagher et al. 2011), insertion-sequencing or INSeq (Goodman et al. 2009), high-throughput insertion tracking by deep sequencing or HITS (Gawronski et al. 2009), and transposon-directed insertion-site sequencing or TraDIS (Langridge et al. 2009), the latter of which has been used to simultaneously phenotype more than a million *Salmonella typhi* mutants in a single pool. In eukaryotes, Roche 454 approaches have been utilized to identify *piggyBac* insertions in mice (Rad et al. 2010); Illumina-based approaches have only rarely been published (Alsford et al. 2011; Rinaldi et al. 2012; Friedel et al. 2013). Here, we describe the development and application of a new high-throughput TraDIS-based method, quantitative insertion-site sequencing (QIseq), which uses Illumina sequencing technology to identify *piggyBac* insertion sites in eukaryotic genomes. For testing, we adapted QIseq to the AT-rich *P. falciparum* genome, but it is applicable to a wide range of eukaryotic genomes.

## Results

### Quantitative insertion-site sequencing

**Figure 1.**
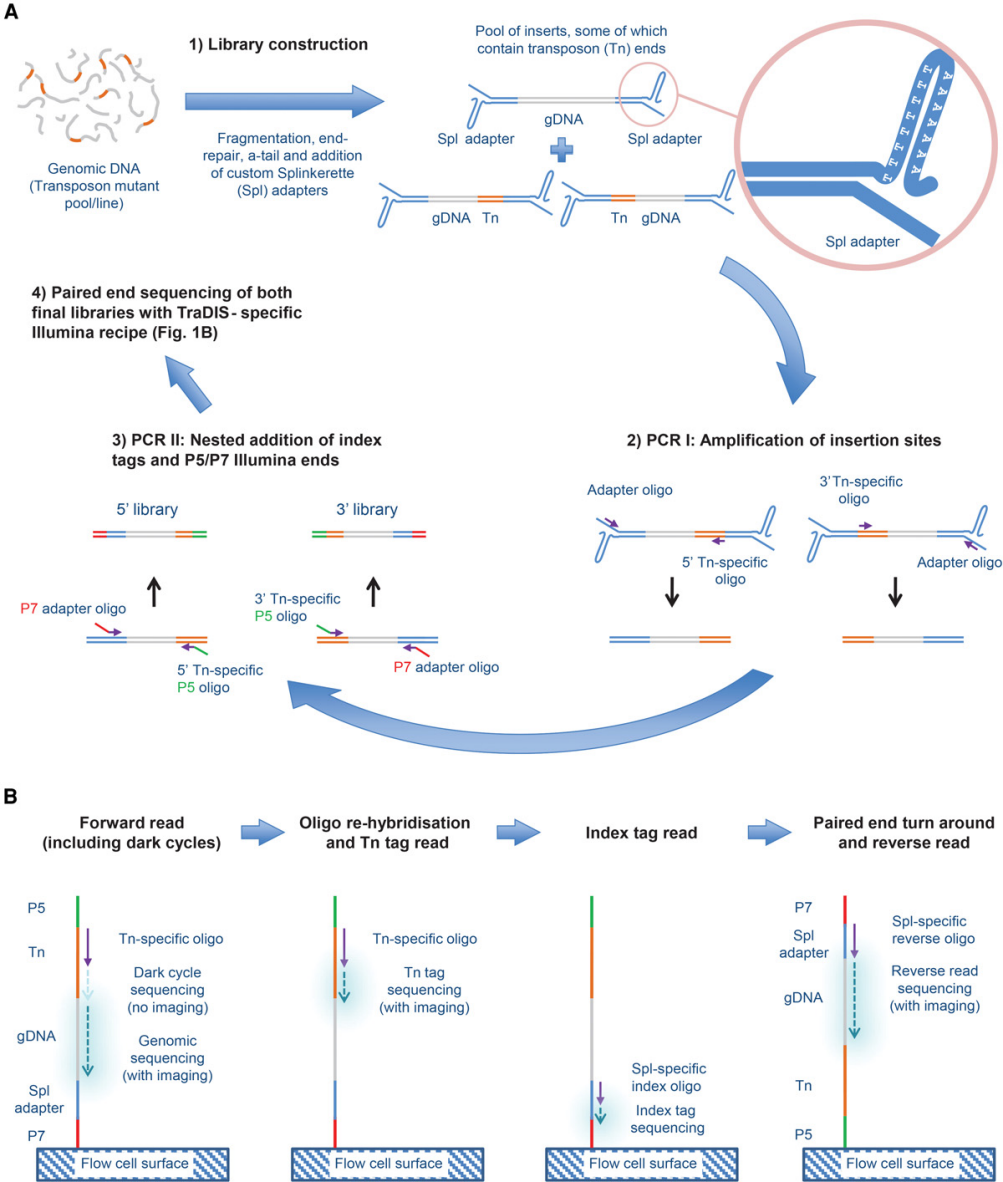
Outline of QIseq process. (*A*) Library generation. (1) Randomly sheared genomic DNA containing *piggyBac* transposon (Tn) insertions is ligated to a custom Splinkerette (Spl) adapter (light blue). (2) Separate 5′- and 3′-Tn-end-specific libraries are generated by linear amplification from the transposon (orange) using 5′- or 3′-specific primers, followed by priming from the adapter. (3) A nested PCR is performed with primers specific to the Tn end and adapter end, containing a P5 (green) and P7 (red) sequence, respectively, to allow the product to bind to the Illumina flow cell for sequencing. (*B*) Sequencing protocol. After binding to the flow cell at the P7 end, a Tn-specific sequencing primer initiates base incorporation. Because all insertion sites will start with the same bases at the end of the Tn, no imaging takes place during the first cycles, with normal imaging initiated only at the genomic DNA sequence. After denaturation, Tn tag sequences are then read using the same sequencing primer, before index tags, which allows sequencing of multiple libraries on one flow cell, and are read using a Splinkerette (Spl)-specific sequencing primer. Finally, a reverse read is generated using a paired-end turnaround, hybridizing the P5 end to the flow cell and sequencing using a Spl-specific reverse oligo.

Quantitative insertion-site sequencing uses Illumina sequencing to recognize and quantify *piggyBac* transposon insertion sites in eukaryotic genomes. As outlined in Figure 1, QIseq relies on customized Splinkerette adapters, which are added to randomly sheared genomic DNA, before transposon insertion sites are specifically amplified using a combination of transposon- and adapter-specific oligonucleotides. Finally, Illumina adapters are added in a nested PCR reaction, and the resulting Illumina library sequenced using either a HiSeq 2500 or MiSeq machine. The method was iteratively developed in order to overcome three major technical challenges: (1) nonspecific background amplification during the PCR steps; (2) monotemplate sequences generated during sequencing (because all reads will begin with the same sequences from the transposon long terminal repeats) interfering with Illumina's template generation step; and (3) the combination of low complexity of the individual clonal libraries combined with the AT richness of the *P. falciparum* genome affecting Illumina's color matrix estimations.

To tackle nonspecific background amplification during the template amplification process, we first optimized library preparation by ligating a custom Splinkerette hairpin adapter, modified from Uren et al. (2009), to sheared, end-repaired, and A-tailed *P. falciparum* genomic DNA. This hairpin adapter avoids mispriming from the adapter's lagging strand during the initial template capture PCR (PCR1) (Fig. 1A) and makes sure that the template for the adapter primer is only generated after a transposon-specific product has been made. Since *piggyBac* transposons are DNA elements with distinguishable 5′ and 3′ inverted terminal repeats (ITRs), PCR1 was performed with two individual (i.e., 5′ and 3′ ITR) *piggyBac* PCR1 primers. This yields two individual products, which allows for the identification of the sequences adjacent to the 5′ and 3′ end of the insertion site, increasing the robustness of insertion site identification. Next, 5′ and 3′ nested amplification was done on these individual pools (PCR2) (Fig. 1A) to increase specificity of the libraries and simultaneously add Illumina flow cell binding P5/P7 ends to the libraries. The two products were separated from adapter dimers and residual primers and molarity determined using quantitative PCR (qPCR).

To distinguish true integrations from putative false positives, ITR-specific sequencing primers were designed 5 bases upstream of the transposon integration site. This meant reads originating from true integration sites would all contain a 5-bp transposon sequence and the *piggyBac* recognition site (TTAA) that positively identifies them as integration sites, but it has the negative outcome that all true transposon reads would start with the same 9 bases. As Illumina sequencers use these first few bases to determine the diversity of the sequencing clusters (http://res.illumina.com/documents/products/technotes/technote_low_diversity_rta.pdf), a modified sequencing recipe was generated from the standard Amplicon recipe (see Supplemental Fig. S1) to omit these bases by performing the first sequencing steps using “dark cycles,” i.e., sequencing cycles where no imaging takes place. While this solved the problem of low diversity, it does not allow the transposon sequence to be read concurrently with the genomic DNA sequence of the insertion site. To confirm that the sequence originated from a transposon integration site, we therefore introduced a second round of sequencing where the initially skipped sequence (containing the transposon sequence) was read by denaturing the template and rehybridizing the same sequencing primer and sequencing these skipped bases as a separate transposon tag read (Fig. 1B).

Due to the AT-rich nature of the *P. falciparum* genome (Gardner et al. 2002), base diversity and therefore color diversity of the clusters is already limited in normal genomic DNA libraries. In our case, this low color diversity was exacerbated further due to a large amount of identical forward reads (monotemplate) originating from clonal transposon-integration sequence. This resulted in incorrect color matrix estimations and anomalous base calling. We therefore added 10%–20% PhiX DNA (i.e., a library of fragments from a well-characterized GC neutral genome) to our QIseq libraries on the MiSeq sequencer to solve this incorrect color matrix calling. The HiSeq 2500, however, has less-advanced real-time analysis (RTA) software, which makes it less able to deal with a monotemplate compared with the MiSeq (results not shown). Furthermore, it will score bases as Ns when no signal is detected in any of the four colors (results not shown). Given these challenges, we added 50% PhiX when sequencing on the HiSeq 2500 platform.

### QIseq accuracy and sensitivity

Having developed the QIseq method, we next investigated accuracy and sensitivity in order to set cut-offs for insertion site identification. A series of *P. falciparum* lines containing *piggyBac* insertions that had been previously cloned and insertion sites identified using inverse PCR (Balu et al. 2009) or whole-genome sequencing were used as test samples. Genomic DNA was extracted from 117 cloned lines, and insertion site identification was carried out by QIseq (Supplemental Tables S1, S2). All clones were in the NF54 strain, whereas the *P. falciparum* reference genome is from strain 3D7. Even though NF54 is the parent strain of the 3D7 clone, the two genomes are not identical, so to ensure more accurate mapping, we assembled an NF54 genome for the purposes of this study (the NF54 and 3D7 genomes differed at <1500 single nucleotide polymorphisms and <500 indels, roughly 10-fold lower than the average level of divergence between any two nonrelated *P. falciparum* lab strains) (see Supplemental Methods S1). Transposon insertion sites were mapped using this new NF54 reference genome, and several publicly available and widely used mapping programs were compared (Supplemental Table S2) before BWA (Li and Durbin 2009) was selected as the most robust for the insertion-site calling pipeline (see Supplemental Material for details of the mapping comparison; the insertion-site calling pipeline is outlined in Supplemental Fig. S1).

**Figure 2.**
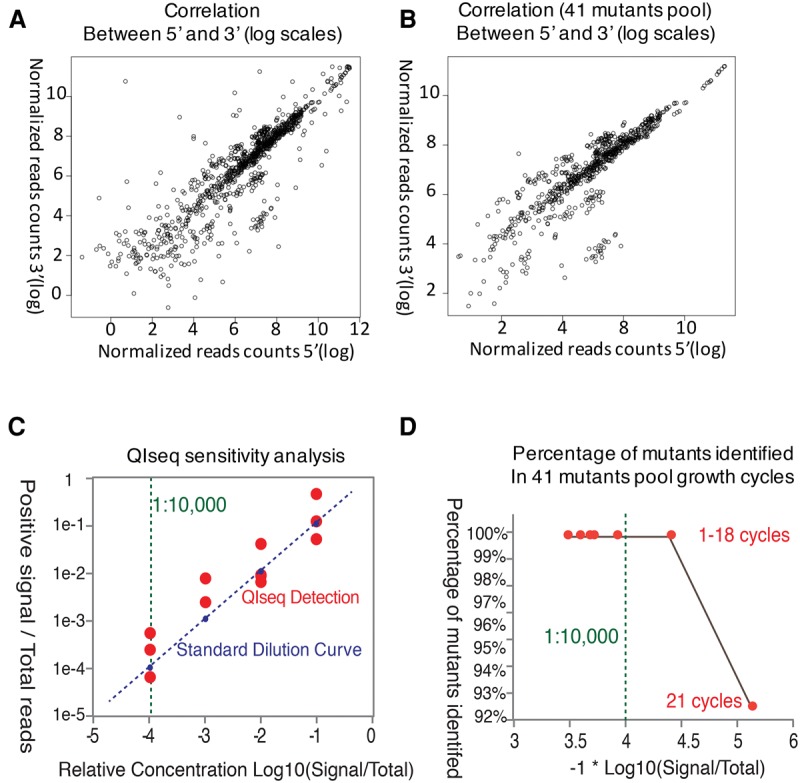
Reproducibility and sensitivity of QIseq. Insertion sites identified during sequencing of multiple single and pooled *piggyBac* lines were compared across biological replicates and time points. (*A*) Correlation plot of insertion sites identified by 5′ and 3′ libraries in small pool of 41 *piggyBac* insertions. (*B*) Correlation plot of the same insertion sites identified in biological replicates. (*C*) QIseq sensitivity analysis of the gDNA of PB-3 mutant line was diluted as 1:10, 1:100, 1:1000, 1:10,000 and mixed with three different mutant lines as biological reps. QIseq identifying and monitoring sites over a >10,000-fold dynamic range of sequence counts. (*D*) Percentage of mutants identified in small pool growth cycles, over a >10,000-fold dynamic range of sequence counts in 18 cycles.

Both 5′ and 3′ QIseq libraries were generated for each sample, and insertion sites were identified in all 117 cloned lines. To determine whether 5′ and 3′ libraries reproducibly identified the same integration sites, correlation plots from individual *P. falciparum* clones were generated. To normalize individual libraries, read quantities were standardized between 5′ and 3′ libraries, and total read-depth was log-transformed. Not only did we observe good correlation between 5′ and 3′ libraries over the full range of integrations in all clones tested (*r*^2^ = 0 0.89–0.99, depending on the data set used), but the slope of all correlations was close to 1 (0.95–1.03) in all cases (Fig. 2A,B). This strong correlation does not, however, rule out the need for using both 5′ and 3′ QIseq libraries to confirm integration sites—while agreement was strong across the whole population, there were inconsistencies between 5′ and 3′ reads in individual cases, perhaps due to specific genomic features such as extreme AT-richness at one side of a given insertion site (Supplemental Fig. S2).

QIseq proved both accurate and sensitive. Of the 117 lines, QIseq identified the same major insertion site as had been identified previously in 113 cases (Supplemental Table S1). In addition, QIseq identified 21 additional *piggyBac* insertion sites in these 117 lines that had not previously been identified by inverse PCR approaches. All of these 21 new insertion sites were repeatedly confirmed in subsequent growth screens described below (Supplemental Table S5) and targeted PCR confirmed the presence of two integration sites in 6/6 lines tested (Supplemental Table S1). While each line had been previously cloned, the presence of two sites in a line could either be the presence of previously undetected double integrations in a single clonal line or the presence of two lines each with a single integration. To distinguish between these alternatives, we recloned two lines (69, 116) by limiting dilution. Targeted PCR confirmed the presence of both QIseq identified sites in these recloned lines, arguing that they do indeed harbor two independent insertions. Overall, QIseq established that at least 87% of *P. falciparum* mutants carried a single *piggyBac* element (Supplemental Table S1). We used the data from the individual cloned lines and also created artificial mixtures of different amounts of DNA from these cloned lines (Fig. 2C) to set detection thresholds for site identification in subsequent pooled approaches (Fig. 2D; Methods; Supplemental Methods S1).

### Identification of insertion sites in uncloned pools

**Figure 3.**
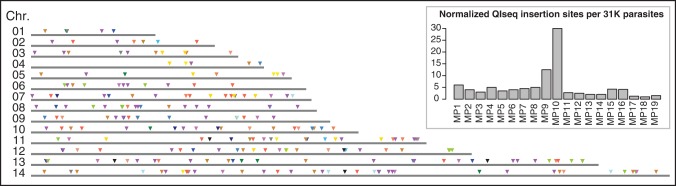
Distribution and frequency of identified *piggyBac* insertion sites in the unknown mixed populations (MPs). Distribution of the 254 QIseq insertion sites of the 19 MPs (Supplemental Table S3) shown along the 14 chromosomes; different colors represent different pools. (*Inset*) Normalized QIseq insertion site: Pools MP1–MP5 were based on 31,000, MP6–MP10 on 62,000, MP11–MP15 on 124,000, and MP15–MP19 248,000 parasites, respectively. The bar plot shows the normalized amount of insertion sites per 31,000 parasites for each unknown MP.

Genetic screens using *P. falciparum piggyBac* mutants could be carried out either with previously cloned lines with known insertion sites that are mixed together and grown in pools, or with pools of unknown insertion sites, such as those generated in a single round of transfection. To test the latter screening scenario, which is the method routinely used in bacterial TraDIS, we used QIseq to identify insertion sites shortly after transfection but before cloning. Ten 96-well microtiter plates, each containing 200 µL of *P. falciparum* NF54 culture per well at a range of parasite concentrations, were transfected with *piggyBac* constructs. These mixed populations (MPs) were grown with drug selection for integration (WR99210 at 2.5 nM) for 5 d, or 2.5 intra-erythrocytic cycles. Roughly half of the MPs survived drug selection, and 19 of these were selected at random for QIseq to estimate how many mutants could be generated in a single round of transfection. QIseq identified 254 *piggyBac* insertion sites across all 19 transfected mixed populations, with the number of insertion sites in each pool varying with the numbers of parasites transfected in each well (Fig. 3, inset; Supplemental Table S3). About half of the tested MPs (47%) contain 8–12 mutants each, with a median value 9 (Supplemental Fig. S3A). Extrapolating this median value across all 450 MPs that were growing after 5 d implies that there were approximately >4000 insertion sites across all transfections in the 10 96-well plates (Supplemental Fig. S3B).

The 254 identified insertion sites were distributed across all 14 chromosomes in a largely random pattern, although two sites, near the ends of Chromosomes 8 and 13, were identified multiple times (Fig. 3). This could potentially be due to index tag contamination, although the filters applied to insertion-site calling makes this unlikely (see Supplemental Methods S1). While 254 insertion sites could be identified that clearly exceeded the QIseq cut-offs, in the majority of transfections only a few sites predominated, with the most common insertion site often identified by 10×–100× more reads than the next most common site (Supplemental Table S3).

### Parallel phenotype analysis of mutant parasite pools

While large uncloned mixed populations of mutants could provide an efficient approach for screening, the large number of potential TTAA insertion sites (>328,000) in the *P. falciparum* genome is likely to result in each population being unique because a different selection of all possible insertion mutants will be generated in each independent transfection. An alternative approach known as parallel phenotyping would create pools of pre-existing *P. falciparum* clones with known *piggyBac* insertion sites, similar to an approach that is now possible using targeted gene modification in *Plasmodium berghei* (Gomes et al. 2015). This approach has the advantage that it can be carried out using a validated library of *piggyBac* clones rather than generating a new pool every time. QIseq could then be used on these known pools to quantitate the relative level of each mutant before and after a given phenotypic selection, in a process similar to what has been done in functional profiling of genomes of other pathogenic eukaryotes (Goranov and Madhani 2014).

**Figure 4.**
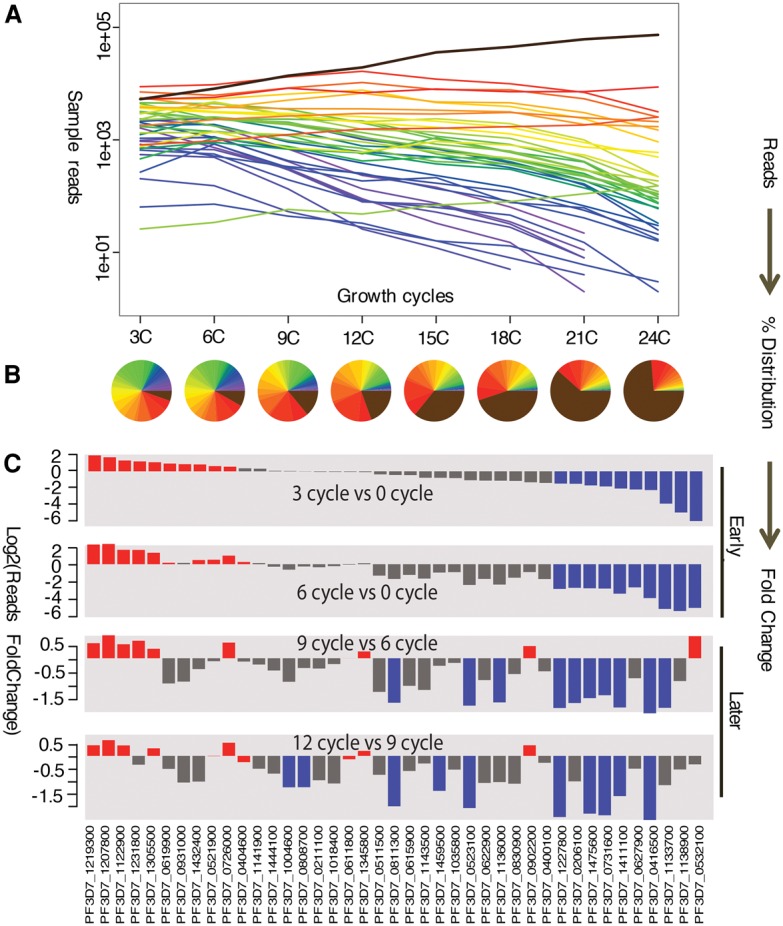
Using QIseq to measure growth of 41 *piggyBac* mutants within a single pool. As described in the Methods, 41 individual *piggyBac* mutants were pooled and grown over 24 cycles, with the relative abundance of each clone measured by QIseq counts every three cycles. (*A*) Growth of each *piggyBac* mutant growth measured by QIseq reads. (*B*) Relative abundance of each *piggyBac* mutant during each measured growth cycle, represented by percentage of reads. (*C*) Identifying neutral or advantageous mutations and deleterious mutations within the pool. Bars represent fold change in read number for each mutant over time; the top quartile (*N* = 10) represents the neutral mutation phenotypes (red) and the bottom quartile represents the deleterious or ‘loser’ mutation phenotypes (blue) in competitive growth conditions.

To test this approach, we evaluated fitness of a small pool created by mixing 41 previously characterized *P. falciparum piggyBac* mutants in equal volumes, and then growing them together in in vitro culture for 24 asexual intra-erythrocytic development cycles, or ∼48 d (Supplemental Table S4). Samples of the small pool, maintained in three biological replicates, were analyzed every three cycles by QIseq to quantify each mutant within the pool (Fig. 4A; Supplemental Table S4). Individual insertion sites for nearly all mutants could be tracked over the entire 24 cycles, emphasizing the remarkably broad range of accuracy in the QIseq methods. At the start of the assay, the relative abundance of each mutant was approximately equal, as expected given that it was created from a roughly equal mix of 41 independent lines. After three asexual development cycles (Fig. 4A), measurable differences in relative abundance were evident, and after 12 cycles, >50% of the mapped reads of the *piggyBac* insertions represented only four mutants (Fig. 4B).

**Figure 5.**
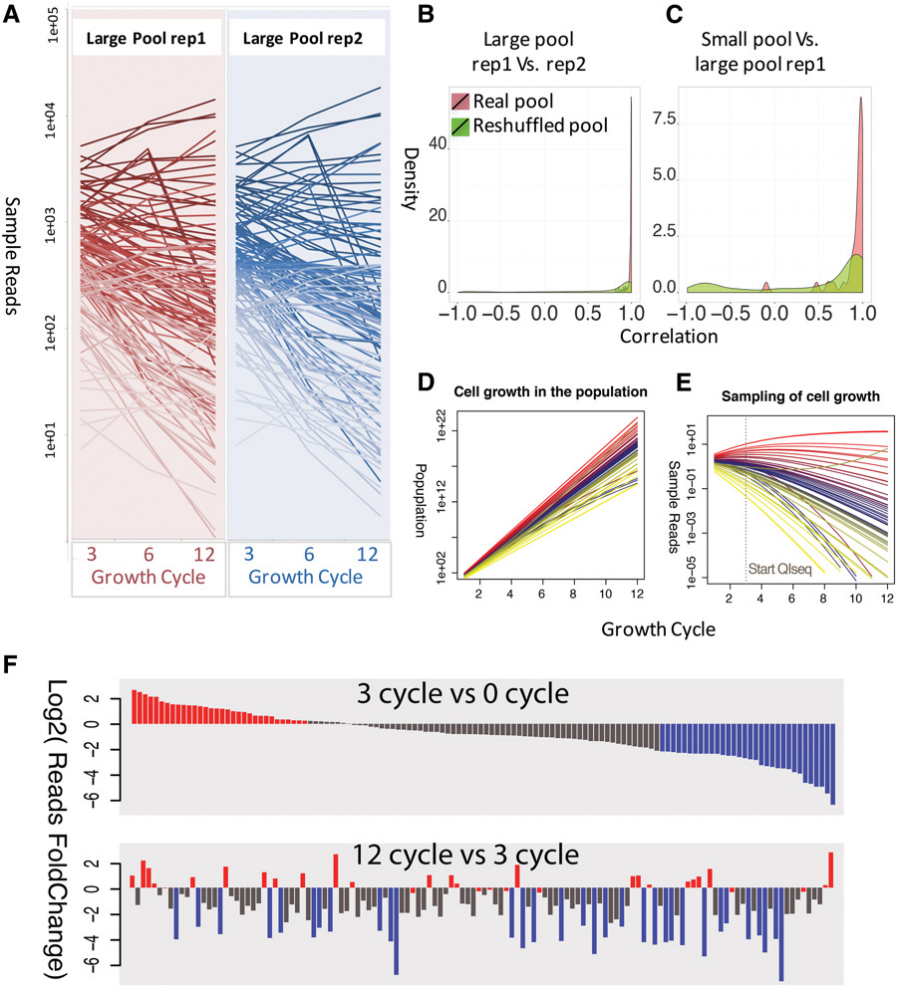
Using QIseq to measure growth of 128 *piggyBac* mutants in a single pool. (*A*) Growth of each mutant within two biological replicates (rep1, rep2) of the same pool, grown over 12 cycles. (*B*) Permutation-based statistical test of reproducibility between replicates and between different screens. (*C*) The same mutants in different replications always had a significantly higher correlation compared with randomly reshuffled mutant pairs (Wilcoxon test *P* < 1 × 10^−5^). (*D*) Estimates of population growth by individual *piggyBac* mutants within the pool of clones grown together over 24 cycles. (*E*) Estimates of QIseq sampling of *piggyBac* mutant populations. While all *piggyBac* mutants continue to proliferate, the relative abundance of most slower growing mutants is predicted to decrease (“bottom quartile”) relative to the few faster growing parasites (“top quartile”). (*F*) Classification of phenotypes from the large pool of 128 mutants identified neutral or advantageous mutations (red; top quartile of growth, *N* = 32) and deleterious mutations (blue; bottom quartile).

To further investigate assay reproducibility, we repeated the competitive fitness assay using two biological replicates of a larger pool of *P. falciparum piggyBac* mutants grown together for 12 cycles (Fig. 5A; Supplemental Table S5). Additional *piggyBac* mutant clones were combined with the first pool to create a larger pool (*n* = 128 insertions), and the process was repeated independently to create two biological replicates. Growth rates measured by QIseq in the large pool replicates were again highly reproducible between replicates consistently identifying >95% of the *piggyBac* insertions in both 5′ and 3′ paired end QIseq (mean Pearson's correlation ≥0.95) (Fig. 5B). Furthermore, there was strong reproducibility between growth rates of mutants in the small pool and also in the larger pool, suggesting that growth rates did not result from experimental error or sampling bias (Fig. 5C). As was the case in the small pool, by 12 development cycles a few clones came to dominate the large pool.

### Identifying phenotypes

**Figure 6.**
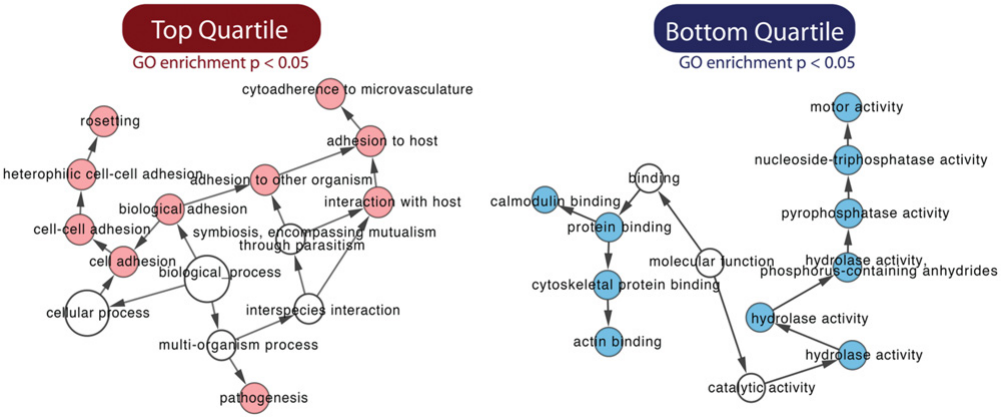
Mutant-associated functions in competitive growth assay for the top and bottom quartiles. The gene enrichment is performed with GO term representation by a hypergeometric test; *P*-value adjusted with the Benjamini-Hochberg method. The solid circle sizes represent the number of mutants in the group.

The patterns of relative change for individual *piggyBac* mutants in each biological replicate of the large pool therefore appear to be highly reproducible. In analyzing these data, it is important to remember that changes of relative abundance determined by QIseq represent a combined measure of a parasite's growth, its proportion in the starting population, and the fixed read number for each Illumina sequencing run. To better understand the relationship between QIseq read number and growth competition among the *P. falciparum* mutants, we simulated the relationship between mapped reads and representative fold change (FC) between growth cycles (Fig. 5D,E). When any two cycles are compared for relative growth phenotype, we classify mutants that rank in the bottom quartile of the pool as having deleterious mutations and those that rank in the top quartile as neutral or advantageous mutations (Fig. 5F). We further distinguish early- vs. late-growth phenotypes, because early phenotypes (Cycle 3) are more likely to represent initial individual growth differences, whereas the late phenotypes (>Cycle 12) also reflect within-system growth competition advantages (neutral mutations) and disadvantages (deleterious mutations). When GO term analysis was performed on the different phenotypes, the genes in the ‘losing’ group were enriched for genes more likely to be critical for growth both in vitro and in vivo, such as protein trafficking, whereas the ‘winning’ group contained an enrichment for pathogenesis genes that are more likely to be neutral in parasite growth in vitro (Fig. 6). Some of the important deleterious mutations occurred in genes encoding ApiAP2 transcription factors, SNF7 vacuolar sorting protein, and upstream of the gene encoding the kelch protein K13 (*K13*, also known as *kelch13*) associated with artemisinin resistance (Ariey et al. 2014). As an independent test, we analyzed the numbers of paralogs of each gene across ten *Plasmodium* species, reasoning that genes with larger numbers of paralogs are more likely to have redundant functions. Genes with growth rates in the bottom quartile of the pool had a significantly lower number of paralog counts than the upper quartile, suggesting that there were fewer gene replacements available to substitute for their function (Supplemental Fig. S4).

## Discussion

We here describe QIseq, a new method for identifying and quantitating *piggyBac* insertion sites in eukaryotic genomes. The method is accurate and sensitive, identifying insertion sites missed by previous approaches. This sensitivity over a wide dynamic range is key for complex biological applications, supporting phenotypic screens of large mixed pools of mutants. While the range could be increased further by increasing sequencing depth, even in these preliminary studies, QIseq could repeatedly identify sites in mixed populations over a 10,000-fold range of sequence counts. A benefit of the high read-depth per integration site is that sequencing costs can be quite moderate, certainly significantly more modest than attempting to identify insertion sites by whole-genome sequencing. In addition, QIseq does not use restriction site cleavage to help verify transposon integration sites, which is used in several other methods to increase specificity for transposon reads (Goodman et al. 2009; Uren et al. 2009; van Opijnen et al. 2009) but does carry the risk of false negatives. The risk of false positives is also reduced because the number of reads from sequences that did not arise from a transposon integration site is reduced by the nested PCR step in QIseq. This is a significant improvement compared to other methods utilizing the Roche 454-sequencing platform (Uren et al. 2009; Rad et al. 2010). One moderate limitation on the depth of reads for *P. falciparum*, and potentially for other AT-rich genomes, is the requirement for large percentages of PhiX on the HiSeq 2500 platform, and to a lesser extent on the MiSeq, in order to prevent the low complexity of the QIseq libraries interfering with sequencing. The addition of PhiX reduces the number of *P. falciparum* reads, so experimenting with reducing the amount of PhiX DNA will be an important development step. Most importantly, QIseq should be applicable to most eukaryotic genomes suitable for use with *piggyBac* and has already been applied to mice to identify oncogenic networks in pancreatic cancer (Rad et al. 2015). Individual adaptations for specific genomes may be required (for example, removing the need to add PhiX DNA for sequencing of QIseq libraries from less AT-rich genomes), but the core principles should be broadly applicable.

Having developed and tested key parameters of QIseq, we applied it to monitoring growth of *P. falciparum piggyBac* mutants in both cloned and uncloned mixed pools. The ability of QIseq to quantitatively identify insertion sites with high sensitivity revealed that small pools of uncloned *P. falciparum* parasites in the first few growth cycles posttransfection contained a median of nine insertion sites per pool. While further technical improvements for transposon activation in *P. falciparum* are clearly needed, the overall number of insertions even at this modest scale was high, with 254 insertion sites identified in 19 mixed populations. Applying QIseq to 450 such MPs, a scale achievable in a single transfection of 10 96-well plates, should therefore yield >4000 insertions. Large-scale transposon mutagenesis of the 5000-gene *P. falciparum* genome could therefore yield saturation with current tools, given sufficient resources and some automation to support the large-scale parasite culture required. Furthermore, insertions were randomly distributed across the 14 chromosomes of the *P. falciparum* genome, emphasizing that *piggyBac* mutagenesis combined with QIseq offers a path for truly random large-scale genetic screening in *P. falciparum*.

How would such screens be carried out? It is likely that strong positive selection approaches will be the most immediately rewarding, such as selecting for mutants with increased resistance to inhibitory compounds (Pradhan et al. 2015) or with the ability to survive environmental stresses such as heat shock. There are two fundamental approaches to performing such screens, either using pools of pre-existing mutants with known insertion sites (“parallel phenotyping”) (Gomes et al. 2015), or by generating new pools for every screen by transfection. Until transposon insertion efficiency is radically improved, the latter approach will be limited, but initial data reported here monitoring growth of up to 128 previously generated mutants in a single pool suggest that the parallel phenotyping approach is immediately tractable. Initial tests of this pooled screening approach showed that growth rates of individual mutants were highly reproducible between biological replicates, and even between pools with different compositions. Within this competitive in vitro environment, mutations in core metabolism-associated genes, such as those involved in protein trafficking, weakened parasites’ competitive fitness, making them losers relative to mutations in genes typically linked to pathogenicity in vivo. The lack of any extreme defect in most of the deficient mutants highlights the importance of phenotype selection, which is typically evaluated only in in vitro monocultures, in defining relative importance or essentiality of a *P. falciparum* gene. Perhaps noteworthy in this regard is the mutant with an insertion in the upstream region of *K13*, which is a gene associated with artemisinin resistance (Ariey et al. 2014). When grown as a clone, this mutant appears to have a growth rate similar to its WT parent NF54 (Balu et al. 2010), but in competition with a heterogeneous mutant population, its relative growth is significantly weaker. This suggests that mutations in this gene can have a competitive growth defect, as has been suggested for other *P. falciparum* drug resistance mutations in vivo. If such a fitness cost for artemisinin resistance does exist in *P. falciparum*, perhaps similar to *pfmdr1* (Hayward et al. 2005), it may explain why artemisinin resistance has spread relatively slowly. Such a finding, coming from a large-scale screen of >100 genes, is exactly the sort of biological insight that QIseq can generate across multiple biological systems.

## Methods

### *Plasmodium falciparum* culture, *piggyBac* mutants, and DNA preparation

*P. falciparum* mutant clones were cultured by standard methods (Moll et al. 2008). *piggyBac* insertions were generated on the NF54 clone background as described previously (Balu et al. 2009, 2010). *P. falciparum* genomic DNA was isolated from late-stage parasites at 3%–5% parasitemia by using a QIAamp DNA Blood Mini Kit (Qiagen). Additional details are provided in the Supplemental Material.

### Quantitative insertion site sequencing

Detailed methods for QIseq library production and sequencing are included in the Supplemental Methods S1. In brief, as outlined in Figure 1, genomic DNA from individual lines of pools was quantified, sheared using an ultrasonicator, and a custom Splinkerette adapter added to all fragment ends. *piggyBac* insertion sites were then amplified using two nested PCR reactions (PCR1 and PCR2), with individual amplifications to amplify both the 5′ and 3′ end of the *piggyBac* transposon, respectively, resulting in two separate libraries for each sample. Library yield was quantified and insertion sites identified using custom Illumina sequencing approaches. The sequencing approach uses specific sequencing primers and a run protocol that incorporated dark cycles and incorporation of PhiX DNA to reduce the impact of monotemplate reads on sequencing acquisition, as well as a second round of sequencing to allow sequencing of the skipped transposon sequence.

### Sequence read processing

The QIseq libraries were sequenced on both a MiSeq and HiSeq 2500, with 5′ and 3′ libraries sequenced separately. Samples were multiplexed. The read length was 75 bp and the fragment size around 350. An overview of sequencing runs and the multiplexing is provided in Supplemental Figure S1. 3′ and 5′ libraries were processed separately, and the reads for each experiment were corrected for possible sequencing errors using SGA, version 0.9.9 (Simpson and Durbin 2012). Reads were first quality trimmed with the function “preprocess” (parameter: -m 51 and --permute-ambiguous) and then corrected with the function “correct” (parameter: -k 51 and -x 3). After trimming, only reads containing the correct QIseq integration sequence (TAGGGTTAANNN for both 5′ and 3′ libraries, NNN is genomic sequence after the TTAA integration site) and their mate pairs were then mapped against the newly generated *P. falciparum* NF54 genome. A custom Perl script ran all the described analysis and summarized the count for each condition, returning the number of insertion sites, their positions, and the number of found reads both 5′ and 3′. Calculation of correlation values and plots were done in R (R Core Team 2016). All scripts were programmed in Perl and bash and are available in Supplemental Methods S2. The QIseq experiments and insertion sites identified are summarized in Table 1.

**Table 1.**
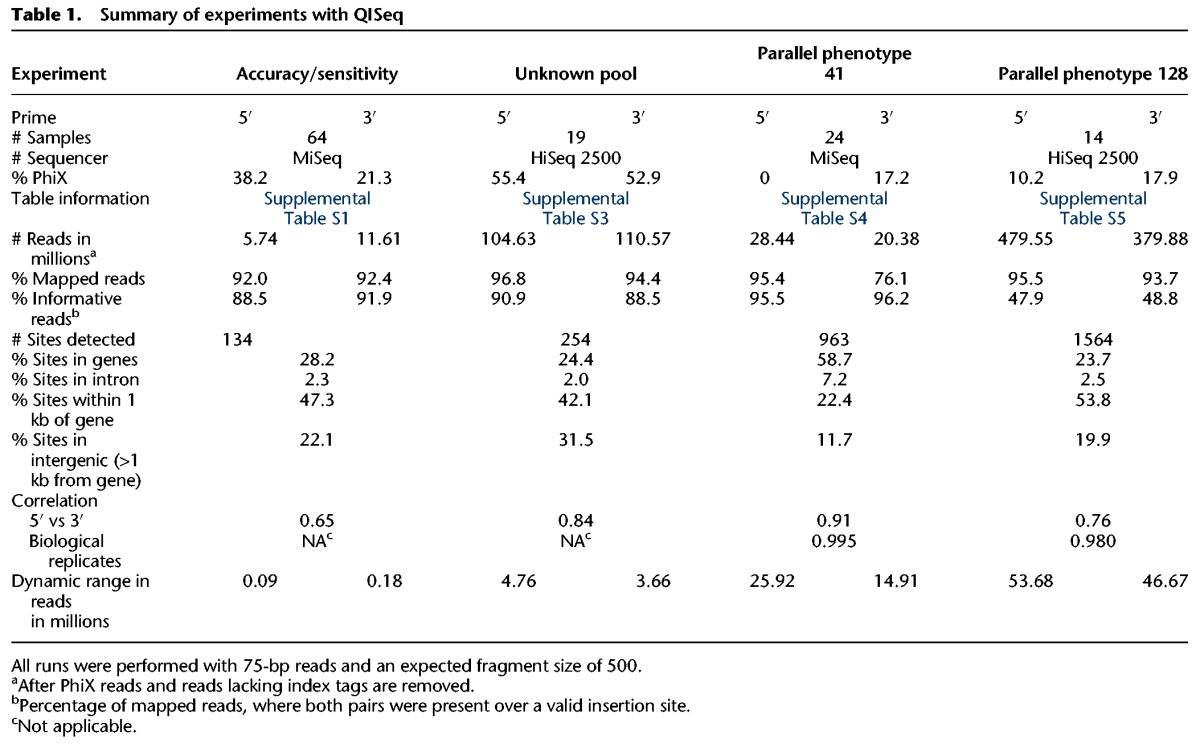
Summary of experiments with QISeq

### Mixed *P. falciparum* mutant pool

*P. falciparum* mutants with known insertion sites were cultured individually. After the cultures grew to 10-mL volume with 2%–3% parasitemia, the cultures were mixed together as a single pool by taking an equal amount of ring-stage parasites. The resulting mixed pools were frozen in multiple aliquots using standard protocols (Moll et al. 2008). Three individual vials of pooled parasites were thawed for use as biological replicates in the growth assay. Pool cultures were grown for 24 generations in 48 d, with sample splitting and dilution when parasitemia levels were high, performed as for standard culture conditions. Samples were taken every three generations and the relative level of each mutant measured by QIseq.

### Analysis of read count correlation between replicates

Bootstrapping was used to estimate the correlation distribution between each sequencing replicate. To rule out the possibility that good correlation between replications can be explained by random permutation of read counts, the read count of each sequencing result was first randomly permutated. Bootstrapping was then used again to estimate the correlation distribution between permutated sequencing replications. The permutation and bootstrapping were repeated 100 times and compared with real sequencing data.

### Analysis of read count correlation between 5′ and 3′

In this analysis, we investigated the read count correlation between 5′ and 3′ normalized QIseq read counts. As shown in Figure 2, A and B, each point represents an insertion, and its read counts in 5′ and 3′ are shown in the horizontal and vertical direction. The Pearson correlation was calculated and shown in Results.

## Data access

All sequencing data from this study have been submitted to the European Nucleotide Archive (ENA; http://www.ebi.ac.uk/ena/) under accession numbers listed in Supplemental Table S6.
